# Extracorporeal Photo-Immunotherapy for Circulating Tumor Cells

**DOI:** 10.1371/journal.pone.0127219

**Published:** 2015-05-26

**Authors:** Gwangseong Kim, Angelo Gaitas

**Affiliations:** 1 Kytaro, Inc., Miami, FL, 33199, United States of America; 2 Florida International University (FIU), Miami, FL, 33199, United States of America; Sapporo Medical University, JAPAN

## Abstract

It is well established that metastasis through the circulatory system is primarily caused by circulating tumor cells (CTCs). In this preliminary effort, we report an approach to eliminate circulating tumor cells from the blood stream by flowing the blood though an extracorporeal tube and applying photodynamic therapy (PDT). Chlorin e6 (Ce6), a photosensitizer, was conjugated to CD44 antibody in order to target PC-3, a prostate cancer cell line. PC-3 cells were successfully stained by the Ce6-CD44 antibody conjugate. PDT was performed on whole blood spiked with stained PC-3 cells. As the blood circulated through a thin transparent medical tube, it was exposed to light of 660 nm wavelength generated by an LED array. An exposure of two minutes was sufficient to achieve selective cancer cell necrosis. In comparison, to PDT of cells growing inside a tissue culture, the PDT on thin tube exhibited significantly enhanced efficiency in cell killing, by minimizing light attenuation by blood. It suggests a new extracorporeal methodology of PDT for treating CTCs as well as other hematological pathogens.

## Introduction

Cancer metastasis is a major culprit for cancer death, given that it is responsible for over 90% of overall cancer deaths [1]. Metastasis occurs through the lymphatic and the circulatory system. During metastasis, some primary cancer cells shed into the blood stream, circulate, and ultimately colonize other organs. These cells classified as, circulating tumor cells (CTC) have a key role in cancer metastasis. A number of studies have focused on detecting, enriching, and enumerating CTCs employing a number of techniques including: micro-fluidic separation devices [2–4], devices that rely on size exclusion by centrifugation [5, 6] or filtration [7, 8], immuno-magnetic separation [9, 10], and fluorescence activated cell sorting (FACS) technologies [2, 11] and several more techniques or combinations thereof. These technologies are diagnostic in nature and are constrained by the small size of blood sample volume.

We hypothesize that removal of CTCs from the blood stream may reduce the chance of metastasis and the aggressive nature of existing tumors [12]. Recent studies report that there is indirect evidence that blood purification procedures, such as hemodialysis, might reduce cancer metastasis and the probability of cancer death by removing circulating tumor cells (CTCs) and other tumor growth factors from the bloodstream [13–15].

Extracorporeal filtration devices using leukocyte depletion filters have been used during tumor surgical procedures to remove tumor cells in order to reduce the risk of their dissemination [13–15], however these devices were not used to reduce metastasis post surgery. There have been efforts to remove or kill cancer cells using microtubes functionalized with antibodies, selectin, and cancer-specific tumor necrosis factor (TNF)—related apoptosis inducing ligand (TRAIL) with a capture and a kill rate between 30–41% [16, 17]. Recently a promising technique involved functionalizing circulating leukocytes with TRAIL and E-selectin adhesion receptor was described [18]. In a recent effort, our group functionalized a simple medical grade tube with human EpCAM antibodies and successfully captured PC-3 cells in whole blood [19].

Here we propose an approach using extracorporeal photodynamic therapy (PDT, or photoimmunotherapy) in conjunction with antibody targeting. PDT requires three components, namely: oxygen, a photosensitizer, and light (mainly in the visible range). All these have to be present at the same time for the photosensitizer to be activated, generate reactive oxygen (principally singlet oxygen O_2_^_^, and damage cells or tissues. Furthermore, the toxicity of the reactive oxygen species is localized to the cell in direct contact with it, due to its short (< 100 nm) diffusion distance [20, 21]. These characteristics result in high specificity to target with near zero collateral damage to adjacent cells/tissues, making PDT an effective and safer treatment compared to conventional radiation and chemotherapy. In spite of these advantages, visible light can barely penetrate through tissue [22, 23], especially in the presence of blood (a visible light absorber) and water (an IR light absorber) and thus the application of PDT is mainly limited to diseases in opened/topical regions, including skin, head, neck, lungs, teeth, etc.

**Fig 1:**
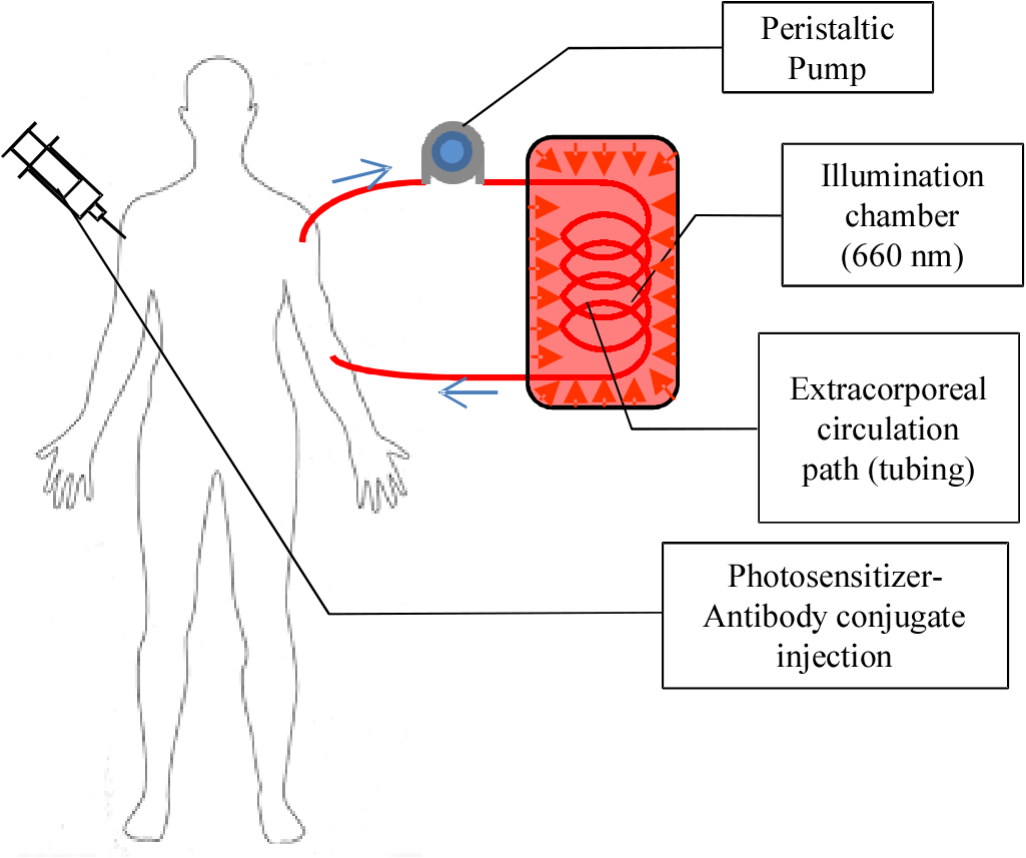
Schematic of the proposed device in operation. Photosensitizer-antibody conjugate is injected prior to PDT procedure. Blood circulation was guided by medical tubing with a peristaltic pump. Extracorporeal PDT is performed as the blood flows through the tube inside a reflective chamber. The treated blood is returned to body. All procedures are accomplished in a constant flow mode.

In this preliminary effort we demonstrated feasibility of a new therapeutic application of PDT for hematological pathogens, by antibody targeting and extracorporeal device to overcome PDT's tissue penetration depth limit. We have developed a photosensitizer (Chlorin E6 (Ce6))—antibody (anti-CD44) conjugate (Ce6-CD44 Ab conjugate) to selectively deliver the photosensitizing agent to CTCs (PC-3 cells in this case). PDT was performed by letting the blood flow through a thin transparent medical tube (Fig 1) exhibited significantly improved cell killing efficacy. An additional benefit to this technique is that the antibody can be safely cleared out of the body by natural antibody degradation mechanisms within a few days [24]. In this work, we demonstrate the proof-of-principle of this approach.

## Materials and Methods

### Conjugation of Ce6 to CD44 antibody

Chlorin E6 (Ce6) (Frontier Scientific) is a naturally occurring, commercially available photosensitizer that has excitation maxima in the far-red/near IR region (around 667 nm) and relatively high quantum efficiency. Because the Ce6 molecule has three carboxyl groups, it can be readily modified for chemical conjugation. Human CD44 antibody (BD Bioscience) was selected for the model human prostate cancer cell line, PC-3 (purchased from American Type Culture Collection (ATCC)). Expression of CD44 in PC-3 cell was previously reported [25] and confirmed experimentally by us. 2 mg of Ce6 was mixed with 6.5 mg of crosslinker, 1-Ethyl-3-[3-dimethylaminopropyl]carbodiimide hydrochloride (EDC) (Sigma-Aldrich) and 7.6 mg of sulfo-NHS (Pierce) in 1 mL PBS buffer at pH 7.4 (at 1:10:10 mole ratio respectively). The reaction ran for 2 hour at room temperature. Then, 50 μL of the product was added to 100 μL of FITC labeled human CD44 antibody solution. The conjugation reaction was run for 3 hours at room temperature with agitation. The reaction mixture was spin-filtered to remove the unbound Ce6 at 4000 RCF for 100 min. The final product was re-suspended in PBS, adjusting the final volume of 100 μL. The produced Ce6-CD44 Ab conjugate was stored at 4°C.

### Cell culture

The PC-3 cell lines were propagated in RPMI media supplemented with 10% fetal bovine serum (FBS) and 1% penicillin-streptomycin (PS). Cells were passaged using trypsin. Cell culture media, trypsin EDTA, and buffers were purchased from Life Technologies.

### Photodynamic therapy setup

**Fig 2:**
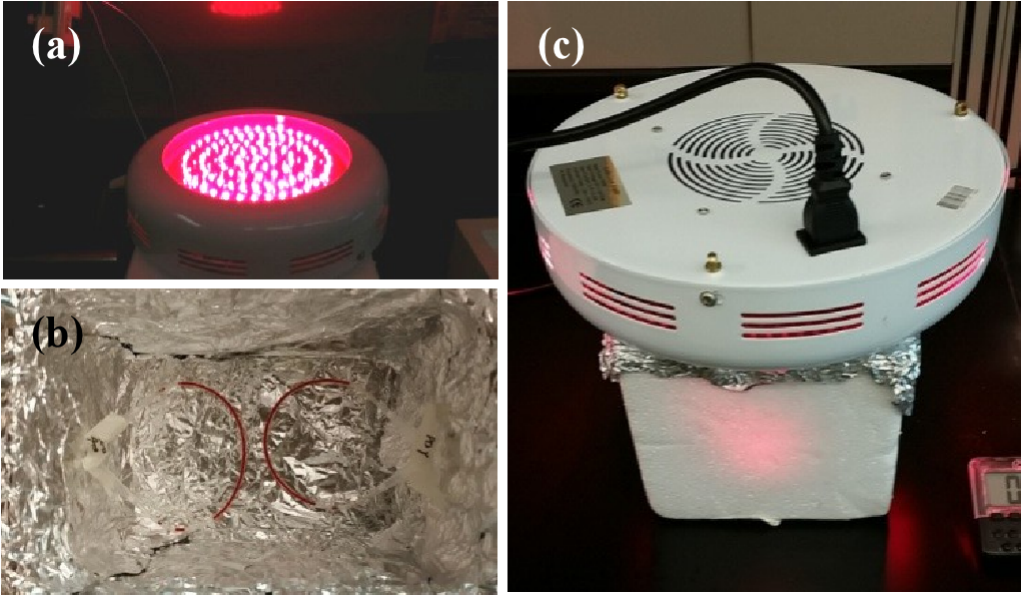
Extracorporeal PDT setup. (a) 660 nm LED array, (b) the aluminum foil covered chamber made from Styrofoam box with two tubes filled with blood. (c) PDT is performed by putting the LED array on top of the chamber. A 2 minute illumination was carried out to simulate extracorporeal blood circulation.

PDT was performed using high power (maximum 100 W input) 660 nm LED array shown in Fig 2(A) (LEDwholesalers.com). Samples were placed within an aluminum foil covered styrofoam chamber shown in Fig 2(B) and Fig 2(C) and illuminated. The duration of illumination was carefully optimized for minimum exposure to PDT treatment.

### PDT on PC-3 cells on a 12-well plate

PC-3 cells were cultured on a 12 well plate. Once the cell culture was confluent, 20 μL of Ce6-CD44 Ab conjugates were added and incubated for 1 hour. The cells were gently rinsed with warm DPBS three times to remove unbound excess Ce6-CD44 Ab conjugates and the plate was filled with 1 mL RPMI medium. The staining of the Ce6-CD44 Ab conjugates was confirmed by the fluorescence of FITC label on CD44 antibody. 10 μL Calcein AM (Life Technologies) was added to the cells and afterwards the cells were incubated for 20 min. The extra Calcein AM was removed by rinsing three times, and the plate was refilled with 0.5 mL warmed whole blood (Innovative Research). PDT was performed by placing the 12-well plate in the PDT chamber and illuminating it for 2 min. The weakening of the Calcein AM florescent signal is an indication of cell death. We monitored the flourescence signal every 15 minutes for 2 hours. Three control experiments were performed in parallel. The first control experiment involved PC-3 cells stained by Ce6-CD44 Ab conjugate in culture media and illuminated by the LED (positive control). The second control included PC-3 cells stained by Ce6-CD44 Ab conjugate without illumination (no light—negative control). The third control consisted of PC-3 cells that were illuminated by the LED but were not stained by Ce6-CD44 Ab conjugate (no conjugate—negative control). The outcome of PDT was monitored by fluorescence imaging with an Olympus IMT-2 inverted microscope connected to a Zenoptik MF Progres camera using Progress Capture Pro software.

### PDT on PC-3 cells in a tube

Confluent PC-3 cells were trysinized using 0.25% trypsin EDTA. The cell were counted using a hemocytometer. We used 300,000 cells / mL for the study. 20 μL of Ce6-CD44 Ab conjugates were added to 1 mL cell suspension and incubated for 1 hour. The cells were pelleted by centrifugation at 1000 RPM for 5 min. The supernatant was removed and the cells were resuspended in 1 mL of fresh RPMI medium. The staining with Ce6-CD44 Ab conjugates was confirmed by fluorescence microscopy based on the FITC label present on the CD44 antibody. The PC-3 cells were stained by Calcein AM for 20 min to monitor cell death. The cell suspension was centrifuged at 1000 RPM for 5 min and the supernatant was removed. The cell pellet was resuspended in RPMI medium to a final density of about 50,000 cells / 20 μL. 20 μL of these cells were spiked into 100 μL of warmed whole blood. After mixing, a 15 cm Silastic polydimethylsiloxane (PDMS, Silicone) tubing (Fisher Scientific) was filled with the PC-3-blood mixture and illuminated for 2 min. The blood specimens were collected. The fluorescence of Calcein AM labeled PC-3 cells was monitored at 0 min (before illumination), and subsequently 1and 2 hours after illumination by leading the cells on a hemocytometer (for counting purposes) placed on the stage of a fluorescence microscope. The reduction in the strongly fluorescing cells was analyzed to determine the efficacy of PDT.

## Results

### PDT on PC-3 cells on a 12-well plate

**Fig 3:**
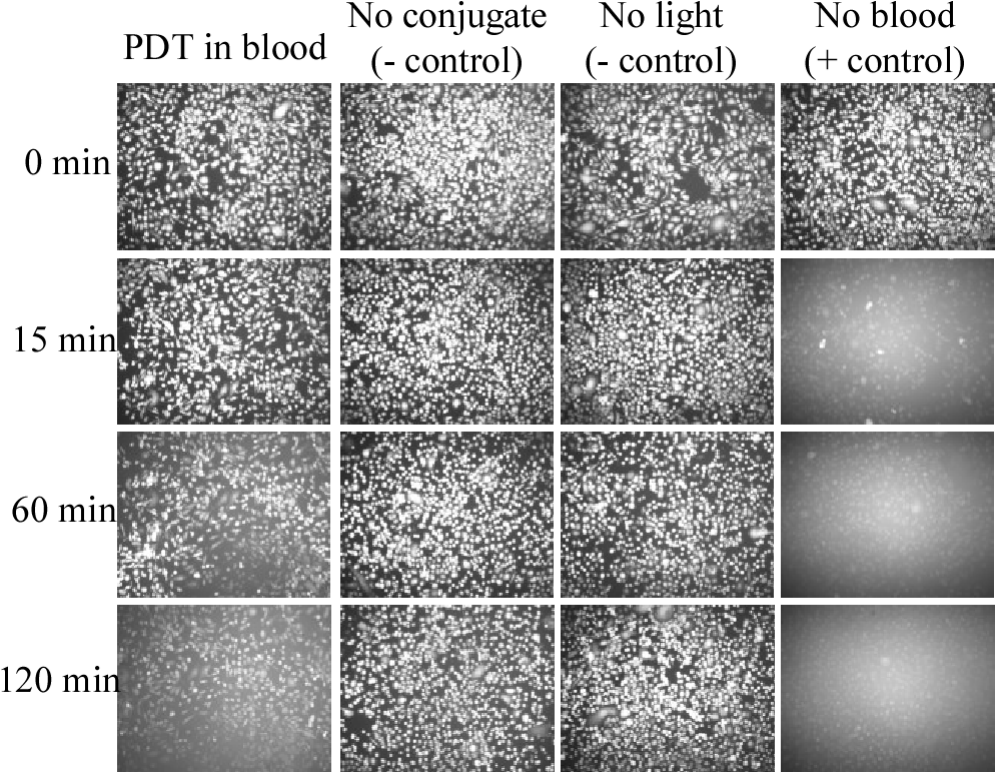
Results of photodynamic therapy after 2 minutes illumination on a TC plate of PC-3 cells. The cells were stained with Calcein AM. The left hand column “PDT in blood” demonstrates diminished cell death with increasing light exposure as compared to the right hand “no blood” column due to light absorption effects by components present in blood. The negative controls do not exhibit any cell death.

PC-3 cells were grown to confluency on the surface of a 12-well plate. After 1 hour of incubation with the Ce6-CD44 Ab conjugate, positive staining was confirmed by monitoring the FITC label on the CD44 antibody (S1 Fig). The cells were stained with a cell viability indicator, Calcein AM. Illumination with the LED was performed in the presence of either 0.5 mL of whole blood or, in the case of the positive control, in 0.5 mL RPMI media. The results are summarized in Fig 3. In the positive control in RPMI media nearly all of the PC-3 cells were killed within the first 15 minutes (in the 15 minutes we are including the 2 minutes of illumination) without any noticeable survival. This result demonstrates that the Ce6-CD44 Ab conjugate is a highly potent photosensitizer reagent with target specificity. However in the presence of whole blood, cell death was considerably slowed down compared to the result in pure cell suspensions and a small population of cells survived after two hours of exposure. The most plausible reason for these results is that hemoglobin in blood is blocking transmission of the light signal to reach the cells growing on the surface of the well bottom of the plate. Both negative controls showed negligible reduction in Calcein AM staining and thus there was no cell death observed.

### PDT on PC-3 cells in a tube

**Fig 4:**
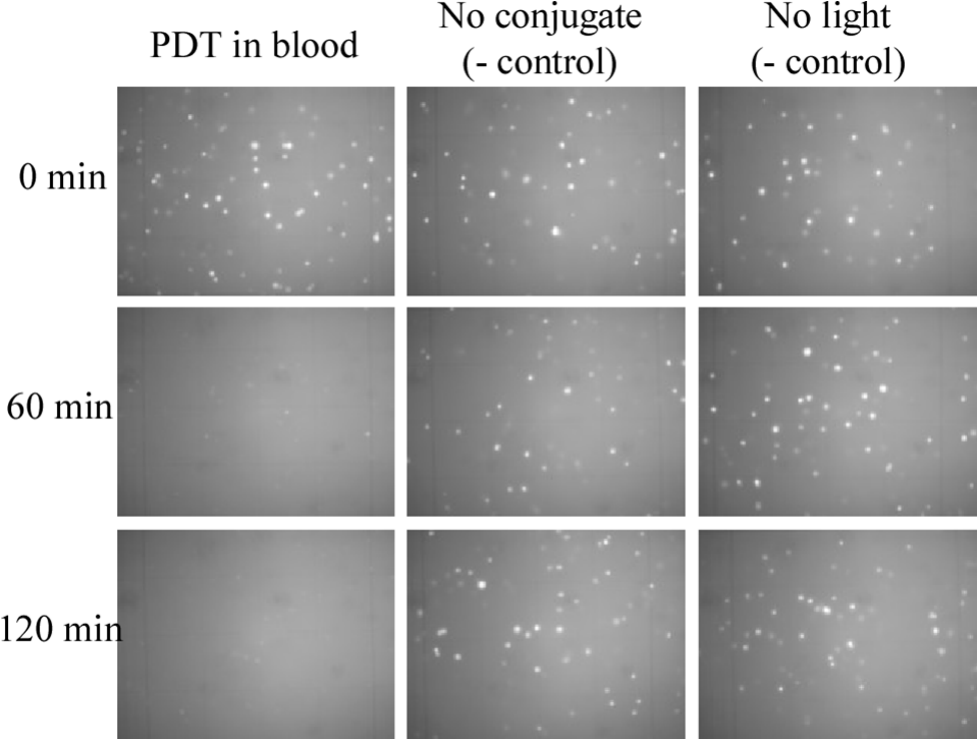
Results of photodynamic therapy in a tube after2 min illumination. The tube’s diameter is 1.02 mm, which is within the range of penetration depth of light given that the tube is illuminated from all directions, for this reason there is accelerated cell death compared to the results in media, as shown in the left hand side “PDT in blood” column.

**Fig 5:**
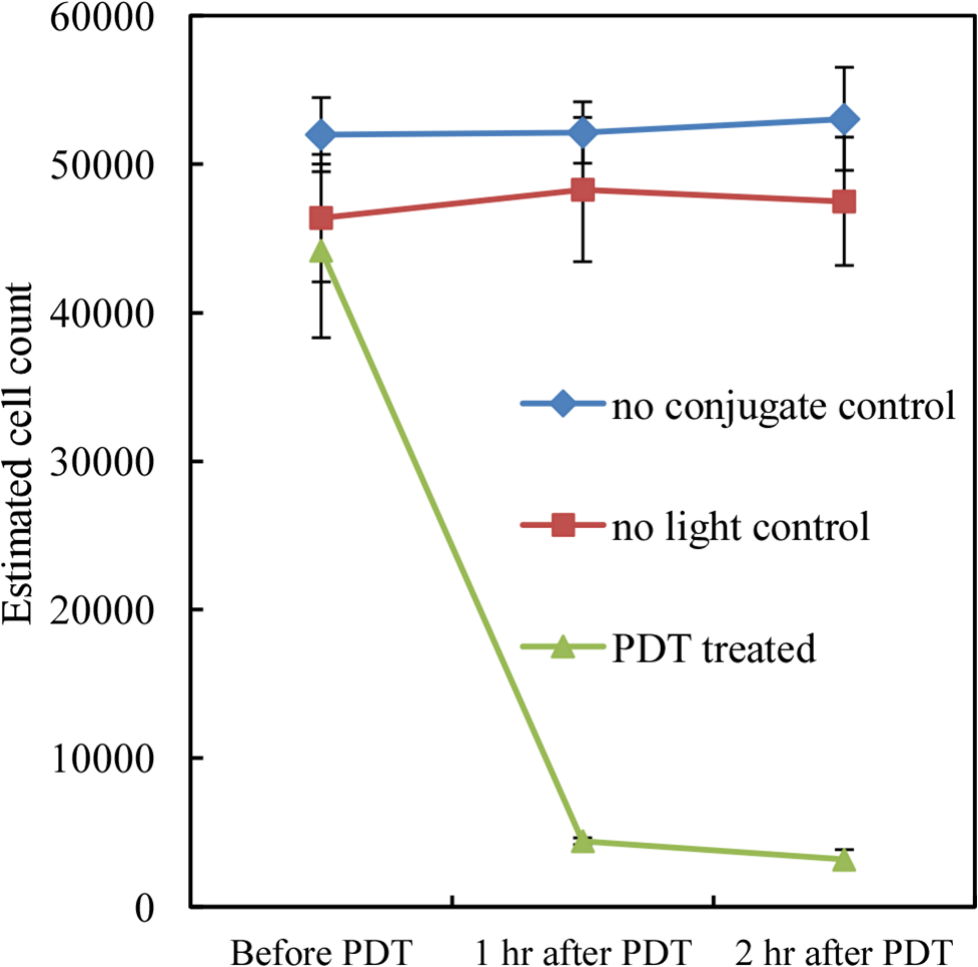
The quantitative analysis of PDT outcome for PC-3 cells in tube. (mean ± SEM) **P<0.*001 PDT treated vs*. *no conjugate control and no light control using t-test*. *N = 3*.

PC-3 cells spiked in whole blood were inserted in a thin PDMS tube to evaluate the efficacy of extracorporeal therapy. About 50,000 PC-3 cells pre-stained with Ce6-CD44 Ab conjugates and Calcein AM were spiked in 100 μL of whole blood. The cell killing effect was studied by monitoring the Calcein AM fluorescence in the presence of blood and also quantified by performing fluorescence imaging. The sample that contained the Ce6-CD44 Ab conjugate, and illuminated by PDT, demonstrated successful cell-death. The samples showed similar initial density in terms of fluorescing PC-3 cells prior to illumination (Fig 4). We monitored cell death after 60 min and 120 min and the results showed significant reduction in fluorescence after the were treated with the Ce6-CD44 Ab conjugate and illuminated (Fig 5). The two negative controls (one with conjugate but without illumination, and the other without conjugate but with illumination) did not exhibit any noticeable change in fluorescence within the 2 hour of observation period (Fig 5). Thus from these observations it is clear that PDT in a tube appears to be more effective in killing cells than in the 12-well TC plate.

## Discussion

We have adequately demonstrated in our preliminary studies the utility of PDT in killing CTCs using a tubing and appropriate antibody binding technology. PDT is an effective alternative treatment modality that addresses several of the drawbacks of conventional treatments in cancer and in other diseases. However, the absorption of visible light by blood (especially due to the red blood cells' hemoglobins) significantly reduces the penetration of light through tissue. This is evident in our results shown in "PDT in blood" column of Fig 3, where PC-3 cells were cultured on a 12-well plate. Following a two minute illumination 2 hours later the many cells were still alive. In contrast, the cells in media were completely wiped out by the two minute illumination in the absence of blood within the first 15 minutes. These results clearly demonstarte that cellular and other components present in blood can hamper efficacy of PDT in killing your target cells.

The results, from Fig 4, where PDT was performed in a tube exhibited improved efficacy. Majority of cell death could be achieved before 1 hour following two minute illumination. We believe that the improvement came from the utilization of the narrow tube that reduced light attenuation by blood. The tube used in this study is a transparent PDMS tube with 1.02 mm inner diameter. Since the light came from all the directions surrounding the tube in the reflective chamber, the thin diameter of tube allowed for nearly the entire sample to be within the penetration depth of light. More exposure to light resulted in better outcomes with PDT. These results promise a new effective treatment modality for hematological pathogens using PDT, overcoming its penetration depth limitation.

The experimental parameters used in this investigation, such as the choice of photosensitizer, the illumination time, the antibody, the type of tube material, size of tube (length and diameter), the light source etc. need further refinement. These parameters need to be adequately addressed and optimized to obtain maximum efficacy of PDT, especially with consideration to *in vivo* constant flow conditions.

A similar extracorporeal approach was suggested previously by Edelson et al. [26] where the "extracorporeal photopheresis (ECP)" concept was first reported. This technology aims to destroy white blood cells in the blood. The fact that this principle is based on UV light and the photosentizer is used without prior targeting, the white blood cells (WBCs) are required to be separated by apheresis to avoid interference by red blood cells (RBCs) and thereby prevent collateral damages to other blood cells. Then the isolated WBCs are treated with UV illumination and re-injected in the body along with other blood components. This technology is used to treat cutaneous T-cell lymphoma by reducing tumor burden and graft-versus-host diseases (GVHD) in organ transplantation by suppressing immune reaction against foreign organs and is currently approved by the FDA. Our present approach has numerous advantages over photopheresis, such as 1) It does nor require time-consuming processes to separate blood cellular components, 2) Our technology achieves active targeting to specific cells of interest and finally 3) Our PDT utilizes light activation in the far-red/near infrared wavelengths that is less harmful and has deeper penetration depth.

In addition, the photosensitizer-antibody conjugates can be used as an imaging contrast to detect metastasized cancers, as well as adapted to other treatment modalities, including endoscopic photodynamic therapy and the ability to target the lymphatic system. and several other diseases. It complements the current approaches that are being used to reduce the number of CTCs in medical centers worldwide.

## Supporting Information

S1 Fig Evidence of positive staining PC-3 lls by Ce6-CD44 Ab conjugate in 12 well plate (left) and suspension (right). (DOCX)
