# Supplementary material for: Extracorporeal Photo-Immunotherapy for Circulating Tumor Cells
Source: PLoS One. 2015 May 26;10(5):e0127219. doi: 10.1371/journal.pone.0127219 (PMC4444246; doi:10.1371/journal.pone.0127219)
Supplement: S1 Fig — (DOCX) [file pone.0127219.s001.docx]

**Supplemental Information**


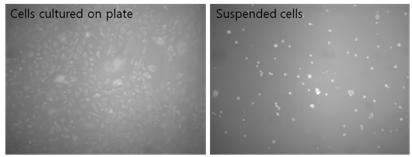


S1 Fig. Evidence of positive staining PC-3 cells by Ce6-CD44 Ab conjugate in 12 well plate (left) and suspension (right).
